# Rotator Cuff Repair with Autologous Fascia Lata Augmentation in Patients at High Risk for Repair Failure: Five-Year Follow-Up Outcomes

**DOI:** 10.3390/jcm15166441

**Published:** 2026-08-20

**Authors:** Sungjoon Lim, Jae-Myeung Chun, In-Ho Jeon

**Affiliations:** 1 Department of Orthopaedic Surgery, CHA Bundang Medical Center, CHA University School of Medicine, Seongnam 13496, Republic of Korea; shoulderdoc82@cha.ac.kr; 2 Department of Orthopaedic Surgery, University of Ulsan College of Medicine, Seoul 05505, Republic of Korea; 3 Department of Orthopaedic Surgery, Asan Medical Center, University of Ulsan College of Medicine, Seoul 05505, Republic of Korea

**Keywords:** shoulder, rotator cuff tear, rotator cuff repair, patch augmentation, fascia lata autograft

## Abstract

**Background**: Large-to-massive rotator cuff tears with advanced fatty degeneration and retears after previous repair remain major clinical challenges. Despite advances in surgical techniques, clinical outcomes remain unsatisfactory. This study evaluated the 5-year outcomes of rotator cuff repair (RCR) with autologous fascia lata augmentation in patients at high risk of repair failure. **Methods**: We retrospectively reviewed 14 patients (mean age, 64.1 ± 6.6 years) who underwent RCR with autologous fascia lata augmentation. All patients had large-to-massive rotator cuff tears with Goutallier grade 3 or 4 fatty degeneration or retears after prior cuff repair. Clinical outcomes were evaluated using the visual analog scale (VAS) for pain, the American Shoulder and Elbow Surgeons (ASES) score, and the Constant score. Postoperative cuff integrity was assessed using magnetic resonance imaging (MRI) or ultrasonography. **Results**: At the 1-year follow-up, significant improvements were observed in pain VAS (from 5.1 ± 1.6 to 1.3 ± 1.0, *p* < 0.001), ASES score (from 51.9 ± 19.1 to 85.2 ± 7.8, *p* < 0.001) and Constant score (from 51.3 ± 13.8 to 70.8 ± 7.0, *p* < 0.001). These improvements were maintained throughout the 5-year follow-up. Postoperative imaging demonstrated an overall retear rate of 7.1% (1/14). No patient underwent revision surgery during follow-up. **Conclusions**: Autologous fascia lata augmentation was associated with sustained pain relief and functional improvement throughout the 5-year follow-up, with a low retear rate on postoperative imaging. These findings suggest that autologous fascia lata may be a viable graft option for rotator cuff augmentation in selected patients, although further prospective comparative studies with larger cohorts are warranted.

## 1. Introduction

Rotator cuff repair (RCR) is among the most commonly performed orthopedic procedures, and its frequency has markedly increased over the past decades [1,2]. Advances in surgical instruments and techniques have broadened indications for repair, while accumulating clinical and imaging studies have improved our understanding of prognostic factors [3,4,5,6]. Among these, tear size and tendon quality are critical biological determinants of repair outcome. Large to massive tears, particularly in the setting of chronic degeneration, are associated with inferior clinical outcomes and high rates of structural failure, with reported retear rates reaching up to 94% after repair of massive tears [7]. Furthermore, outcomes following revision repair are generally less favorable than those of primary repair, highlighting the challenges in managing this high-risk population [8,9,10].

To improve outcomes in these high-risk patients, various biologic strategies have been explored to enhance tendon healing. Platelet-rich plasma (PRP), a widely used biologic adjunct in orthopedic practice, and bone marrow stimulation (BMS) techniques have shown inconsistent clinical benefits, particularly in large to massive tears [11,12,13,14]. These findings underscore the need for more reliable strategies that provide both mechanical reinforcement and a biologic scaffold to support tendon healing.

To address these challenges, patch graft augmentation has emerged as a promising strategy in challenging rotator cuff repair. This technique involves placing a biologic or synthetic graft over the repaired tendon to reinforce structurally vulnerable regions. By reducing mechanical stress at the repair site and providing a scaffold, patch grafts aim to promote tendon-to-bone healing and improve structural integrity [15,16,17]. However, available graft materials differ in their biological incorporation, mechanical properties, and graft-related complications.

Grafts used for patch augmentation can be broadly categorized into xenografts, allografts, autografts, and synthetic materials. Early xenografts, particularly those derived from porcine small intestine submucosa, were largely abandoned because of high failure rates and adverse host responses [18,19]. Allografts, most commonly acellular dermal matrix (ADM) derived from human dermis, are widely utilized because of their ease of use and clinical availability [20]. Synthetic grafts have also been explored but concerns regarding long-term durability and foreign-body reactions have limited their widespread adoption [21,22,23,24]. In contrast, autologous grafts offer inherent advantages, including excellent biocompatibility, absence of immunologic risk, and favorable mechanical properties, making them a potentially reliable option for biologic augmentation in high-risk rotator cuff repair.

Despite these advantages, clinical evidence supporting autologous augmentation remains limited, particularly with mid- to long-term follow-up. Among autologous options, fascia lata has several theoretical advantages, including excellent biocompatibility and favorable mechanical properties. Therefore, the purpose of this study was to evaluate the clinical and structural outcomes of rotator cuff repair with autologous fascia lata augmentation in patients at high risk for repair failure at a minimum 5-year follow-up.

## 2. Materials and Methods

### 2.1. Patient Selection

This retrospective study was approved by our institutional review board (IRB No. S2021-1168). Consecutive patients who underwent rotator cuff repair with autologous fascia lata augmentation at a tertiary university hospital between March 2017 and February 2018 were identified from the institutional surgical database.

The indications for augmentation surgery included large to massive posterosuperior rotator cuff tears in patients with poor biological factors. Large tears were defined as tears greater than 3 cm, and massive tears as those greater than 5 cm or involving complete disruption of at least two tendons [25,26]. Poor biological factors were defined as advanced fatty degeneration (Goutallier grade 3–4) or a history of failed prior rotator cuff repair (revision cases). All tears were considered reparable based on intraoperative assessment.

Anterosuperior cuff tears predominantly involving the subscapularis were excluded, as they represent a distinct tear pattern with different mechanisms and clinical outcomes. Reparable subscapularis tears limited to the upper one-third (Lafosse type I or II) [27] were included when present in association with large to massive posterosuperior tears. Patients with radiographic evidence of advanced glenohumeral joint arthritis, defined as Hamada grade ≥3 on standard anteroposterior shoulder radiographs, were excluded [28].

### 2.2. Surgical Technique

**Figure 1:**
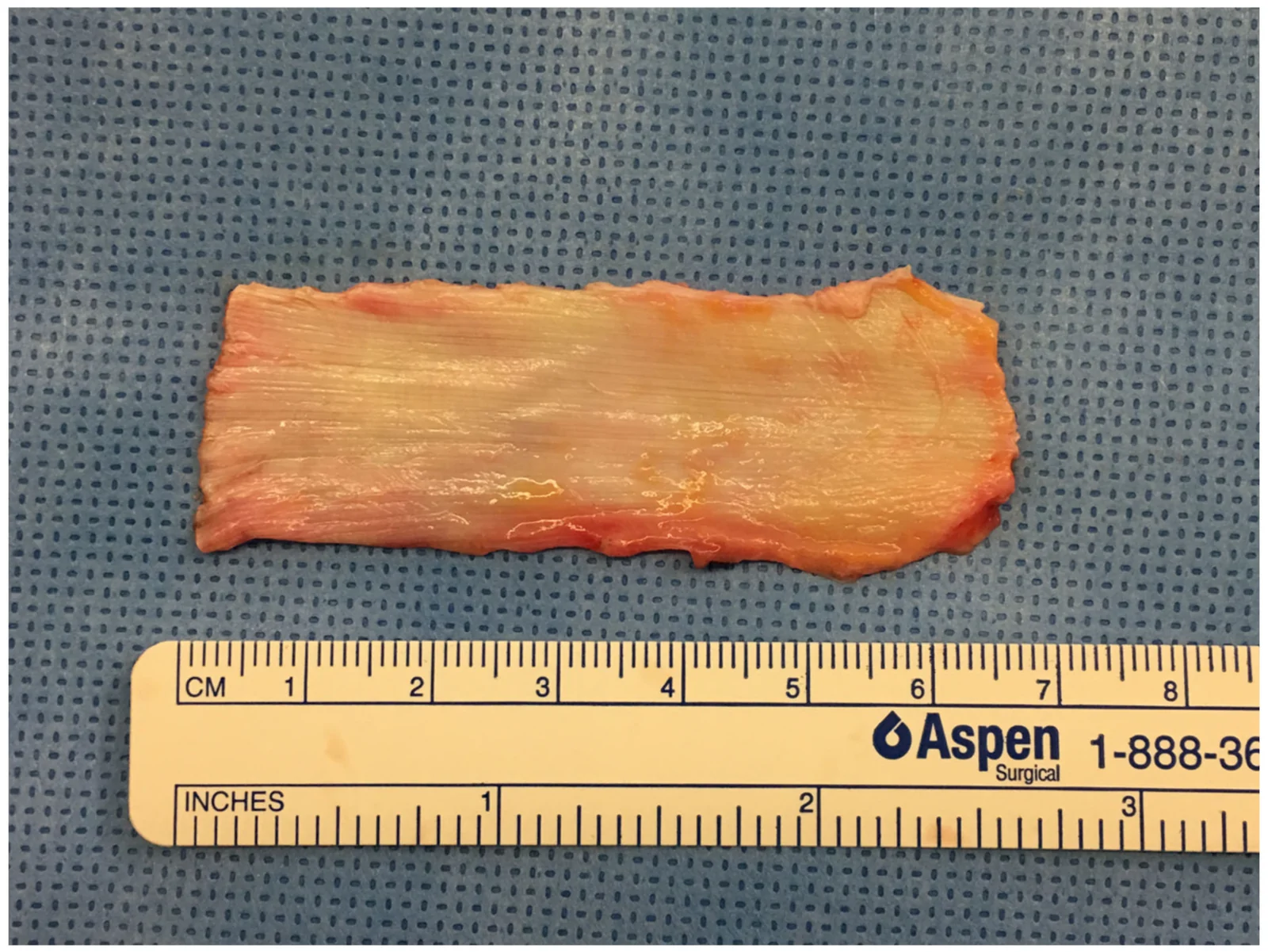
Harvested autologous fascia lata graft measuring approximately 3 × 6 cm.

**Figure 2:**
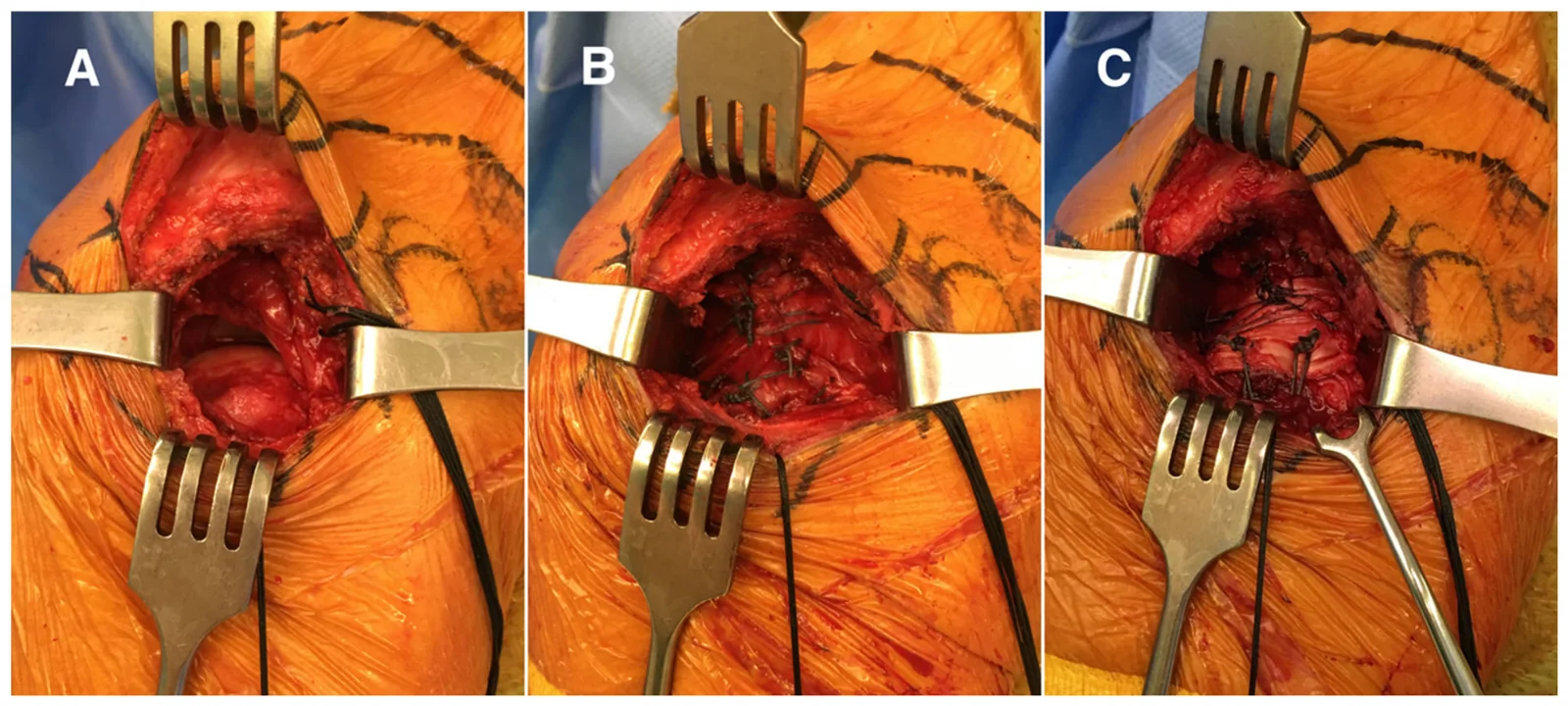
Surgical procedure for autologous fascia lata augmentation. (**A**) Massive cuff tear involving the supraspinatus and infraspinatus tendons. (**B**) Rotator cuff repair performed using multiple transosseous sutures. (**C**) The fascia lata graft was folded into a double-layer construct and securely sutured over the repaired rotator cuff.

All procedures were performed by a single senior shoulder surgeon. An ipsilateral fascia lata graft measuring approximately 3 × 6 cm was harvested (Figure 1). After graft harvest, rotator cuff repair was performed through a standard open anterosuperior approach. Acromioplasty was performed selectively when a subacromial spur was present. The torn cuff was mobilized, and reparable subscapularis tears, when present, were repaired prior to posterosuperior cuff repair. When necessary, additional releases were performed to achieve sufficient tendon mobilization for coverage of the greater tuberosity footprint. The rotator cuff was reattached to the greater tuberosity using transosseous sutures. The fascia lata graft was folded into a double layer and placed over the repaired cuff, then secured with multiple nonabsorbable sutures (Figure 2). No additional biologic adjuncts, including PRP or bone marrow stimulation, were used.

### 2.3. Postoperative Rehabilitation

Postoperatively, the shoulder was protected in an abduction brace for 8 weeks. Pendulum exercises were initiated at 2 weeks postoperatively, followed by passive range-of-motion exercises beginning at 4 weeks. Progressive strengthening of the rotator cuff and periscapular muscles was initiated at 12 weeks postoperatively.

### 2.4. Imaging Study

Preoperative magnetic resonance imaging (MRI) was used to assess tear size and fatty degeneration, which was graded according to the Goutallier classification [29,30]. Postoperative tendon integrity was evaluated at 12 months using a 3.0-Tesla MRI (Achieva, Philips Medical Systems, Amsterdam, The Netherlands). For patients who declined postoperative MRI, musculoskeletal ultrasonography was used as an alternative imaging modality. A retear was defined as the presence of a full-thickness defect or discontinuity at the repair site. The acromiohumeral distance (AHD), defined as the vertical distance between the undersurface of the acromion and the superior margin of the humeral head, was measured on standard anteroposterior shoulder radiographs preoperatively and at annual follow-up [31]. All imaging studies were independently reviewed by a fellowship-trained shoulder surgeon who was not involved in the patients’ clinical care and who was blinded to clinical outcomes.

### 2.5. Clinical Evaluation

Patients were followed up at 1, 3, 6, and 12 months postoperatively and annually thereafter. Clinical evaluations were performed by a single experienced nurse practitioner to ensure consistency. Active range of motion, including forward elevation and external rotation, was measured using a goniometer. Pain was assessed using the visual analog scale (VAS), and functional outcomes were evaluated using the American Shoulder and Elbow Surgeons (ASES) score and the Constant score. Postoperative complications, including surgical and donor-site–related events, and revision surgery were assessed through medical record review.

### 2.6. Statistical Analysis

Continuous variables were presented as mean ± standard deviation. Changes in clinical outcomes across the preoperative and postoperative follow-up time points were assessed using the Friedman test. When a significant overall difference was identified, post hoc comparisons between preoperative and each postoperative time point were performed using the Wilcoxon signed-rank test with Bonferroni correction. The Wilcoxon signed-rank test was used for paired comparisons of preoperative and postoperative acromiohumeral distance. Statistical analyses were conducted using SPSS software (version 21; IBM, Armonk, NY, USA), with statistical significance set at *p* < 0.05.

## 3. Results

During the study period, 15 patients underwent rotator cuff repair augmented with autologous fascia lata. One patient was lost to follow-up, leaving 14 patients (mean age, 64.1 ± 6.6 years) for final analysis. Baseline demographic and clinical characteristics are summarized in Table 1.

Preoperative MRI demonstrated mean tear sizes of 32.9 ± 8.3 mm in the anteroposterior (AP) dimension and 33.9 ± 5.3 mm in the mediolateral (ML) dimension. All tears involved both the supraspinatus and infraspinatus tendons, and 9 cases were classified as massive tears due to complete disruption of both tendons. Two patients had full-thickness tears of the upper one-third of the subscapularis. The mean preoperative Goutallier grade was 0.9 ± 0.6 for the subscapularis, 3.1 ± 0.3 for the supraspinatus, and 2.8 ± 0.8 for the infraspinatus.

**Figure 3:**
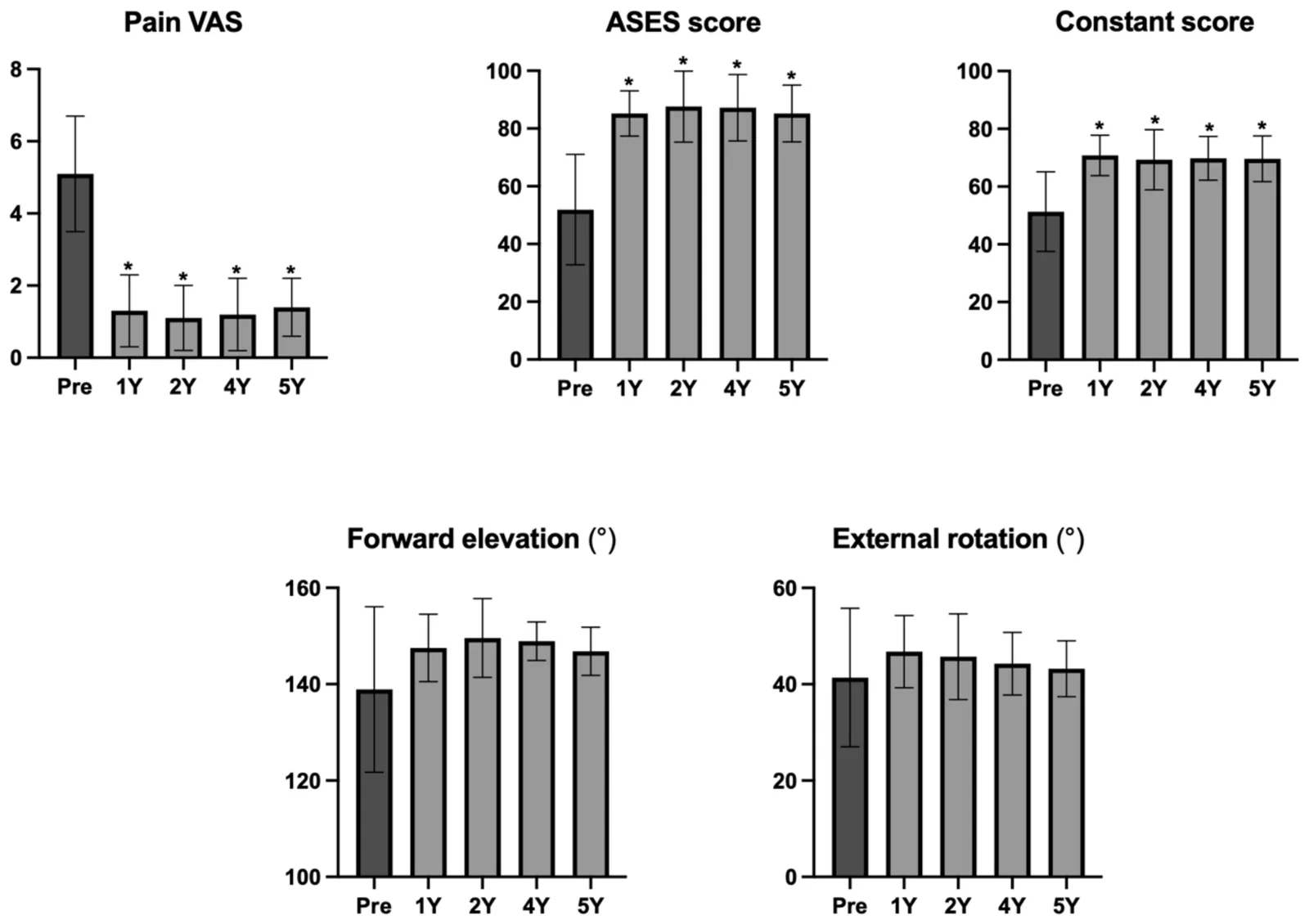
Changes in clinical outcomes over time. Values are presented as mean ± SD. An asterisk (*) indicates a significant difference from the preoperative value based on post hoc Wilcoxon signed-rank tests with Bonferroni correction (*p* < 0.0125). Three-year follow-up data were unavailable due to the COVID-19 pandemic.

Baseline pain VAS, ASES, and Constant scores were 5.1 ± 1.6, 51.9 ± 19.1, and 51.3 ± 13.8, respectively. Pain VAS, ASES, and Constant scores improved significantly over time (overall *p* < 0.001, <0.001, and 0.004, respectively), with post hoc analyses demonstrating significant improvements from preoperative values at all postoperative follow-up time points. These improvements were maintained over 5 years. No significant changes over time were observed in forward elevation or external rotation (overall *p* = 0.239 and 0.438, respectively) (Table 2, Figure 3). Three-year follow-up assessments were not available due to restrictions during the COVID-19 pandemic. No surgery-related complications or reoperations were observed during the follow-up period.

**Figure 4:**
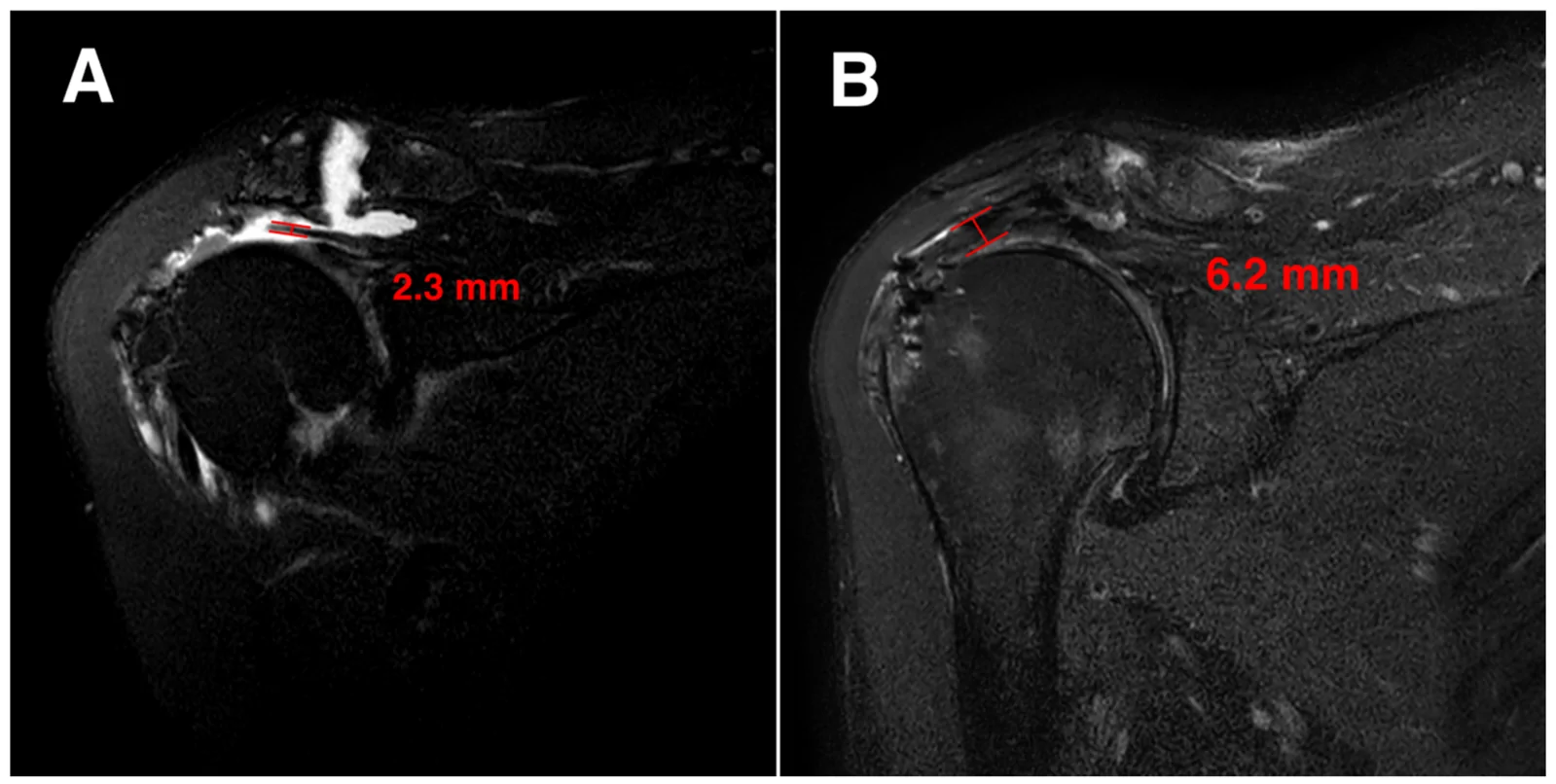
Magnetic resonance imaging of a 67-year-old male with a massive cuff tear of the right shoulder. (**A**) Preoperative coronal MRI demonstrating a tendon thickness of 2.3 mm at the thinnest portion of the supraspinatus tendon. (**B**) Postoperative MRI obtained 12 months after surgery demonstrating a healed tendon with increased thickness (6.2 mm) at the corresponding location.

Postoperative MRI at 12 months was available for 11 patients, revealing one retear. For the remaining 3 patients who declined MRI, musculoskeletal ultrasonography was performed, and no retear was detected. Overall, the retear rate was 7.1% (1 of 14). The mean AHD increased significantly from 5.5 ± 2.0 mm preoperatively to 8.4 ± 1.3 mm postoperatively (*p* < 0.001). A representative case is shown in Figure 4.

## 4. Discussion

The most important finding of this study is that rotator cuff repair with autologous fascia lata augmentation resulted in sustained clinical improvement and a low retear rate over a 5-year follow-up in patients at high risk of repair failure. Despite the challenging nature of this cohort, only one retear (7.1%) was observed, and no patient required revision surgery during follow-up. In addition, no clinically significant donor-site morbidity was identified. These findings suggest that autologous fascia lata augmentation may be a viable option in selected high-risk patients.

The favorable clinical outcomes observed in the present study were characterized by substantial improvements in pain and functional outcome scores that were maintained throughout the follow-up period. In contrast, no significant changes in shoulder range of motion were observed over time. The relatively preserved preoperative forward elevation in our cohort (mean, 138.9°) may have limited the potential for further improvement. Preexisting degenerative changes in the rotator cuff muscles and tendons, together with structural and biomechanical alterations associated with the surgical reconstruction, may also have limited recovery of shoulder motion despite successful tendon healing. These findings suggest that the favorable postoperative outcomes observed in this cohort were characterized primarily by improvements in pain and functional scores rather than by gains in range of motion.

Structural failure remains a major concern following repair of large-to-massive rotator cuff tears, particularly in patients at high risk for repair failure. Previous studies have suggested that failed tendon healing may compromise postoperative clinical outcomes, supporting the rationale for biologic augmentation in these challenging cases [32,33]. Accordingly, patch augmentation has emerged as a promising adjunct by providing both mechanical reinforcement and a scaffold for tendon healing, with previous experimental and clinical studies demonstrating reduced retear rates and improved functional outcomes in high-risk repairs [34,35,36,37]. However, uncertainty remains regarding the optimal graft material and the long-term durability of augmentation techniques. While most published studies have focused on xenografts and allografts, long-term clinical evidence for autologous augmentation remains limited. Therefore, the present study provides long-term clinical and radiologic data on autologous fascia lata augmentation in patients at high risk for repair failure.

Among the various graft materials, xenografts were initially introduced because of their availability and theoretical biological advantages. However, early xenografts, particularly porcine small intestine submucosa (SIS), were largely abandoned because of immunologic complications and unsatisfactory clinical outcomes [18,19]. More recently, porcine-derived acellular dermal matrices have shown encouraging short-term results, with several studies reporting improved tendon healing and functional outcomes following patch augmentation [38,39,40]. Nevertheless, concerns regarding graft-associated inflammation and the lack of long-term clinical evidence continue to limit widespread acceptance of xenografts [41].

In contrast, allografts, particularly acellular dermal matrix (ADM), have become widely used because of their reduced immunogenicity, ease of handling, and clinical availability. However, several concerns remain, including high cost, variability in graft processing and biologic properties, and potential host inflammatory reactions [36,42,43,44]. Although complications after rotator cuff repair with ADM appear to be uncommon, adverse host reactions related to ADM scaffolds have been reported in orthopedic applications [36,43]. These limitations continue to raise questions regarding the long-term reliability and cost-effectiveness of ADM augmentation.

Autologous grafts offer several inherent advantages over xenografts and allografts, including the absence of immunologic risk and favorable biologic and biomechanical properties. The long head of the biceps tendon has been explored as an autologous augmentation material. However, its applicability may be limited in patients with large-to-massive tears because of tendon degeneration, rupture, or insufficient tissue volume [45,46]. In contrast, fascia lata provides a structurally robust, flat graft with favorable biologic and biomechanical characteristics for patch augmentation. Experimental studies have demonstrated enhanced collagen remodeling, reduced inflammatory response, and superior biomechanical properties with fascia lata compared with acellular dermal matrix augmentation. These findings support the potential role of fascia lata as an autologous augmentation material in high-risk rotator cuff repair [47,48].

Fascia lata has long been used as a graft material in a variety of reconstructive procedures [49]. In shoulder surgery, it has also demonstrated favorable long-term outcomes in superior capsule reconstruction for irreparable rotator cuff tears [50,51]. Although additional operative time and donor-site morbidity remain potential disadvantages, no clinically significant donor-site complications were observed in the present study. Compared with superior capsule reconstruction, rotator cuff augmentation requires a substantially smaller fascia lata graft, which may partly explain the low donor-site morbidity observed in our cohort. Together with the absence of additional graft costs, these findings support autologous fascia lata as a practical graft option in appropriately selected patients.

The present study adds to the limited literature by reporting 5-year clinical and radiologic outcomes after rotator cuff repair with autologous fascia lata augmentation [48,52]. Despite the challenging nature of the study cohort, patients demonstrated sustained improvements in pain and shoulder function, with a low retear rate throughout follow-up. In addition, no clinically significant donor-site morbidity, infection, or revision surgery was observed. Taken together, these findings provide valuable long-term data and support further investigation of autologous fascia lata augmentation in larger comparative studies.

This study has several limitations. First, its retrospective design may have introduced selection bias and unaccounted confounding factors. Second, the small sample size limits statistical power and generalizability, and the absence of a control group precludes direct comparison with other graft augmentation strategies. The inclusion of both primary and revision repairs also introduced cohort heterogeneity. Third, although structural outcomes were assessed using magnetic resonance imaging, not all patients underwent postoperative MRI, potentially introducing assessment bias. In addition, the final clinical and imaging follow-up time points were not identical, limiting the availability of long-term structural assessment. Future prospective comparative studies with larger patient cohorts are warranted to further validate these findings.

## 5. Conclusions

In patients at high risk of repair failure, autologous fascia lata augmentation was associated with sustained pain relief and functional improvement throughout the 5-year follow-up, with a low retear rate on postoperative imaging. No clinically significant donor-site morbidity, major postoperative complications, or revision surgery was observed. These findings suggest that autologous fascia lata may be a viable graft option for rotator cuff augmentation in selected patients. Further prospective comparative studies with larger patient cohorts are warranted to better define the role of autologous fascia lata in rotator cuff augmentation.

## Figures and Tables

**Table 1:** Baseline characteristics of the study cohort.

No.	Age (y)	Sex	Side	Tear Size (AP × ML, mm)	Goutallier Stage (SSC/SSP/ISP)	Risk Factors
1	59	F	Rt	35 × 43	1/3/2	Previous RCR
2	69	F	Rt	29 × 34	1/3/2	None
3	61	M	Rt	42 × 25	2/3/2	DM, Previous RCR
4	76	F	Lt	30 × 35	1/3/3	HTN
5	67	M	Rt	38 × 32	2/3/2	None
6	70	F	Rt	35 × 33	1/3/3	HTN
7	60	F	Rt	36 × 39	1/3/4	HTN
8	73	M	Rt	45 × 38	0/3/3	HTN, Arrhythmia
9	67	M	Rt	16 × 30	0/3/2	None
10	59	F	Rt	30 × 31	1/4/4	Hyperlipidemia
11	63	F	Rt	30 × 35	1/3/3	None
12	60	F	Lt	20 × 33	1/3/3	HTN, Previous RCR
13	51	M	Lt	31 × 25	0/3/2	None
14	62	M	Lt	44 × 41	1/3/4	HTN

Abbreviations: AP, anteroposterior; ML, mediolateral; SSC, subscapularis; SSP, supraspinatus; ISP, infraspinatus; RCR, rotator cuff repair; DM, diabetes mellitus; HTN, hypertension.

**Table 2:** Clinical outcomes after rotator cuff repair with autologous fascia lata augmentation.

Variable	Preop	1 yr	2 yr	4 yr	5 yr	Overall *p*
Pain VAS	5.1 ± 1.6	1.3 ± 1.0 *	1.1 ± 0.9 *	1.2 ± 1.0 *	1.4 ± 0.8 *	<0.001
ASES score	51.9 ± 19.1	85.2 ± 7.8 *	87.6 ± 12.3 *	87.2 ± 11.5 *	85.2 ± 9.8 *	<0.001
Constant score	51.3 ± 13.8	70.8 ± 7.0 *	69.3 ± 10.4 *	69.8 ± 7.6 *	69.6 ± 7.9 *	0.004
Forward elevation (°)	138.9 ± 17.2	147.5 ± 7.0	149.6 ± 8.2	148.9 ± 4.0	146.8 ± 5.0	0.239
External rotation (°)	41.4 ± 14.4	46.8 ± 7.5	45.7 ± 8.9	44.3 ± 6.5	43.2 ± 5.8	0.438

Values are presented as mean ± standard deviation. Overall *p*-values were determined using the Friedman test across all time points. An asterisk (*) indicates a significant difference from the preoperative value based on post hoc Wilcoxon signed-rank tests with Bonferroni correction (*p* < 0.0125). Abbreviations: VAS, visual analog scale; ASES, American Shoulder and Elbow Surgeons.

## Data Availability

The data are not publicly available because they contain information that could compromise patient privacy.
